# Healthcare Supply Chain Reliability: The Case of Medical Air Transport

**DOI:** 10.3390/ijerph19074336

**Published:** 2022-04-04

**Authors:** Beata Skowron-Grabowska, Marta Wincewicz-Bosy, Małgorzata Dymyt, Adam Sadowski, Tomasz Dymyt, Katarzyna Wąsowska

**Affiliations:** 1 Faculty of Management, Czestochowa University of Technology, 42-200 Czestochowa, Poland; b.skowron-grabowska@pcz.pl; 2 Faculty of Management, General Tadeusz Kosciuszko Military University of Land Forces, ul. P. Czajkowskiego 109, 51-147 Wrocław, Poland; marta.wincewicz-bosy@awl.edu.pl; 3 Institute of Logistics and Informatics, The University of Lodz, 90-214 Lodz, Poland; adam.sadowski@uni.lodz.pl; 4 T. Marciniak Lower Silesian Specialist Hospital—Emergency Medicine Centre, ul. Gen. A. E. Fieldorfa 2, 54-049 Wrocław, Poland; tdymyt@wp.pl; 5 Institute of Management and Quality Service, Siedlce University of Natural Sciences and Humanities, ul. Konarskiego 2, 08-110 Siedlce, Poland; katarzyna.wasowska@uph.edu.pl

**Keywords:** medical air transport, supply chain, reliability factors, transition of care, decision-making, supply chain mapping

## Abstract

The principal task of national healthcare systems is to provide health services that are safe, accessible, high-quality and people-oriented. To ensure the continuity of healthcare, support activities related to patient transfer and logistics are necessary. Effective implementation of transport processes is a factor affecting the possibility of medical intervention, in terms of both planned and life-saving care. The reliability of the healthcare supply chain is a key factor in patient health. In our research, we have used the method of a single case study of a public regional hospital in Poland providing comprehensive medical services. The aim of the research is to identify the factors that affect the reliability of the healthcare supply chain in relation to the interhospital air transport of patients. Our qualitative research using process mapping reveals what factors affect the reliability of interhospital medical air transport. The analysis of 100 interhospital medical air transport cases has allowed us to create a general procedure related to the movement of patients between the facilities of the healthcare system in Poland. Our research shows that the key factor of reliability is the coherent and integrated cooperation of institutions involved in transport processes.

## 1. Introduction

One of the fundamental tasks of healthcare systems is the efficient delivery of health services. In a well-functioning health system, the provision of services should have the following features: comprehensiveness, accessibility, coverage, continuity, quality, person-centeredness, coordination, accountability and efficiency [1]. High-quality health services ensure that the right care is delivered at the right time, responding to the needs and preferences of service users, while minimising damage and waste of resources [2].

Striving to ensure the continuity of healthcare involves the need for patients to flow within the health system. Patient transfer is carried out in two forms: a primary form, where the patient is transferred from the place of injury or illness to the hospital, and a secondary form, where the patient is transferred to another hospital [3]. Safe patient flow, in terms of both primary and secondary transfers between hospitals, requires efficient logistics based on cooperation within the partner network of entities participating in the provision of health services [4].

Patient logistics as a field in the area of operations and supply chain management covers all planning and control decisions aimed at matching supply and demand throughout the entire healthcare supply chain, in particular the coordination of activities between healthcare organisations [5].

The healthcare supply chain is defined as information, supplies and finances related to the acquisition and flow of goods and services from the supplier to the end user to enhance clinical outcomes while controlling costs [6]. Due to the specificity of health services, the supply chain is not limited to physical goods (drugs, pharmaceuticals, medical devices, health aids, etc.) but also concerns the flow of patients [7]. Turhan and Vayvay [8] indicate that management of the healthcare supply chain is different from other industries due to its tendency for misalignment, high costs for healthcare providers and heavy dependence on third parties. The healthcare supply chain is decentralised, characterised by a lack of coordination mechanisms (financial or contractual) among physicians, hospitals and patients, and is subjected to significant regulatory pressure [9].

The functioning of such a system is threatened by various disruptions; therefore, due to the specificity of health services, it is particularly important to focus on the reliability of medical supply chain management at the strategic and operational levels.

Reliability is the most important feature of logistics processes carried out in the field of patient transport within the healthcare system. However, the consideration of healthcare supply chain reliability varies depending on the context in which the patient transfer takes place. It is considered differently for victims of road accidents [10] and transfers between health system facilities. The reliability of the supply chain through which the flow of patients, medical staff, information, and material goods takes place is vital in ensuring the continuity of healthcare in the field of planned medical interventions and in life-saving emergencies. The aim of this research is to identify the factors that shape the reliability of the healthcare supply chain in the case of patients transported by medical air transport between hospitals. The main motivation for conducting the research was a real problem reported by the employees of the HOSP1 hospital. It refers to the current qualitative procedure for ensuring the continuity of patient care in inter-organisational relations, in complex situations where at least three entities take part in the process. The procedure in force in the hospital was of a general nature and required the clarification of competence resulting from process implementation for individual entities and persons.

Our research, unlike previous studies, which analysed medical air transport in situations of imminent threat to the life and health of patients, presents patient flows between facilities, such as hospitals, in the healthcare system [11,12,13,14]. Our research fills the research gap in the area of reliability of supply chain management by identifying critical factors for the reliability of air medical transport in the flow of patients between hospitals. It should be noted that the few previous studies on medical air transport between hospitals did not focus on a comprehensive presentation of the course of the patient flow process. Instead, they concerned individual types of clinical cases such as strokes, extracorporeal membrane oxygenation, COVID-19 patients and transport physiology [15,16,17,18]. The novelty of our research is a holistic view of patient flow reliability, covering the different clinical characteristics of hospital patients directly affecting the requirements for interhospital movement. They are important because findings are based on the approach of real case evidence research.

Moving patients by means of medical transport between hospitals requires multilateral coordination between the receiving and releasing medical facilities, as well as Medical Air Rescue (MAR). Although this transport is planned, in this healthcare supply chain, there is uncertainty related to the patient’s state of health. It disrupts patient flows between hospitals and blocks the necessary resources in the form of available means of air transport.

In our research, we have used the single case study method by analysing the healthcare supply chain of the HOSP1 public hospital (fictitious name) located in the Lower Silesian Voivodeship in Poland. We chose this hospital because of its access to the hospital’s internal data and processes. Polish regulations on medical air transport are based on EU (European Union) regulations and guidelines of EHAC (European HEMS and Air Ambulance Committee), of which the Medical Air Rescue is a member. Consequently, the context of our observations is broader than national. We conducted our research using various methods, such as expert interviews, document analysis, procedure analysis, procedure mapping, participatory observation, analysis of legal acts and analysis of directives and quality standards.

The complexity of the healthcare supply chain associated with the high dynamics of changes in the patient’s health means that all logistics processes must be characterised by the highest reliability. In addition, logistics processes must comply with applicable medical procedures in accordance with national regulations.

Based on the theory of supply chain management, we have formulated two research questions:

1. What factors affect the reliability of the supply chain in the case of interhospital medical air transport?
2. To what extent does the triangulation of methods allow us to create a general procedure for interhospital medical air transport?

The specificity of the operation of hospitals corresponds to the standards of the High Reliability Organisation (HRO) created by the World Health Organization (WHO). Therefore, acting in an appropriate manner can contribute to providing a highly predictable, safe and effective operation, taking into account the simplification and standardisation of operational tasks, as well as anticipating and protecting against organisational disruptions [19]. Sanders points out that the organisational culture of HRO, which is based on the awareness of risk, the acknowledgement of uncertainty and the recognition of mistakes, should enable and cultivate effective, ethical communication inside and outside the organisation, which in turn increases the trust and involvement of stakeholders and citizens [20].

Striving for reliability means a concentration of activities such as engaging leadership in building a culture of safety services (zero harm to the patient), the inclusion of all principles and practices of safety culture throughout the organisation, and the widespread adoption and implementation of the most effective tools and methods for improving processes [21].

The remaining part of the article is organised as follows. Section 2 provides an overview of the medical air transport literature in the context of healthcare supply chain reliability. Section 3 discusses the methodology and material used in the research. The following parts contain the results of the research carried out and a critical discussion on the reliability of the interhospital medical air transport. The final section contains the conclusions of the conducted research.

## 2. Literature Review

### 2.1. Emerging Topics in Healthcare Supply Management

Healthcare supply chain management is very fragmented. Each stage of the supply chain retains a high degree of independence, which leads to disruptions in the operation of this integrated system. Additional cost pressure while simultaneously ensuring high-quality medical services reinforces the risk of disruption. This applies to all components of the healthcare supply chain, which is the hospital’s main service. These include catering, laundry, maintenance, cleaning and patient transportation [22].

The efficient operation of supply chains requires integrated strategic management. The management approach in relation to the participants of the supply chain in the healthcare system must be oriented towards quality, safety and fundamental values related to health. The concept of supply chain management in general means “*the management of upstream and downstream relationships with suppliers and customers in order to deliver superior customer value at less cost to the supply chain as a whole*” [23] (p. 13).

According van der Vorst [24] (p. 6), supply chain management (SCM) means “the integrated planning, co-ordination and control of all business processes and activities in the supply chain to deliver superior consumer value at less cost to the supply chain as a whole whilst satisfying requirements of other stakeholders in the supply chain (e.g., government and NGO’s)”. As emphasised by Sweeney [25], SCM refers to the systemic and strategic coordination of traditional business functions and tactics within individual entities, their functional units and the supply chain, aimed at improving the long-term results of individual companies and the entire supply chain.

Currently, supply chain management research is focused on many different problems. This includes lean management and sustainability [26], information architecture [27] and performance measurement [28].

The state of the art in HSCM (Healthcare Supply Chain Management), like SCM, includes several leading research paths based on the latest trends in supply chain management. These include issues of supply chain resilience, risk management, patient safety, implementation of modern information technologies and the lean thinking approach [29,30,31,32,33,34,35,36]. Dixit, Routroy and Dubey [37] presented future research directions in the health supply chain in a systematic review of the literature. They drew attention to such SC areas as operations, performance measurement, inventory management, lean and agile operation and IT in many research studies, as well as employee and customer training, tracking and visibility of medicines, cold chain management, HR practices, risk management and waste management. At the same time, we agree that in many of these areas, little research has been carried out so far.

The complexity and dynamism of healthcare supply chain management involves special challenges, such as the lack of consolidation at the provider level, policy issues related to the regulatory authorities, lack of coordination and cooperation between supply chain streams (upstream and downstream), fragmented and inconsistent operation, other chain participants and a high degree of individualisation of activities [38]. In the context of the above, improvement of the healthcare supply chain healthcare is associated with the use of the concept of the logistics chain as a system of transport and warehouse relations, allowing for the development of process aspects that are components of the logistics system. Research confirms that healthcare supply chain performance and agility are influenced by the quality of human capital, both medical and logistic [39].

Therefore, it is necessary to strive for reliability, which is deemed the most important feature of the logistics process [40]. The concept of reliability is defined as the “probability that a system or a product will perform in a satisfactory manner for a given period of time when used under specified operating conditions” [41]. Reliability is the ability of an item (any component, system or subsystem that can be considered an entity) to perform the required function (necessary to provide a specific service), under given environmental and operational conditions for a stated period of time [42].

In healthcare, reliability means failure-free operation over time and is related to effectiveness (where failure may result from not applying evidence), timeliness (where failure results from not taking action within the time required) and patient-centeredness (where failure results from non-compliance with values and preferences of patients) [43]. In relation to the logistics system, reliability is the ability to meet the customer’s demand through the supply chain in which the material flow process is undisturbed by the possible unreliability of suppliers (chain links) [40]. In general, supply chain reliability is defined as the ability to meet customer requirements [44] and expectations in terms of logistics performance [45].

In another approach, reliability means the probability that the chain will fulfil the mission requirements by providing the necessary supplies to the critical transfer points in the system [46]. Due to the specificity of the health system, the reliability of the supply chain in which logistic processes related to patient flow are carried out mainly relates to satisfying the patient’s needs in ensuring healthcare continuity.

In the logistics process of patient transfer, the following actions are necessary: making a decision about the transfer and communication, stabilising and preparing the patient for the transfer, choosing the appropriate mode of transfer (land or air transport), involving the staff accompanying the patient, protecting equipment monitoring the patient’s condition during the transfer, preparation of documentation and transfer of the patient to the receiving institution [47]. While making decisions regarding in-hospital transport, several determinants should be assessed, in particular, patient characteristics, indications for transport, level of escort and transport facilities [48].

The complexity of this process has a significant impact on supply chain coordination, and it consequently determines patient safety. Maintaining patients’ safety and providing them with a highly advanced level of care in the complex and dynamic air ambulance environment requires dealing with serious life threats and rapidly evolving medical issues without the support and facilities available in a hospital environment [49].

### 2.2. General View on Medical Air Transport in Poland

Therefore, these issues are strictly regulated by law. There are also accreditation institutions in the Polish healthcare system authorised to set standards and conduct certification activities in the field of procedures implemented and carried out by hospitals and other entities of the healthcare system. In accordance with the law in force in Poland—the Act of 27 August 2004 on healthcare services financed from public funds [50]—the task of the air transport team is to perform sanitary transport, including transport to the nearest therapeutic entity providing services to the appropriate extent, and back, in two cases: (1) the need for immediate medical treatment in a therapeutic entity and (2) the need to maintain continuity of treatment. Conditions for performance of guaranteed medical rescue services provided by the air medical rescue team are specified in the Regulation of the Minister of Health of 4 February 2019 on guaranteed services in the field of emergency medical services [51] and the Act of 8 September 2006 on the State Emergency Medical Services [52]. In terms of these legal conditions, requirements for medical staff and team members, equipment and medical devices, and team and communication requirements were specified. The air emergency team consists of at least three people, including at least one professional pilot, a system doctor and a system paramedic or nurse.

The medical air rescue team is equipped with a specialised means of sanitary transport meeting the technical and quality features defined in the standards. The communication requirements include: (1) having radio communication ensuring communication of the air medical rescue team with the medical dispatch office, hospital emergency departments, trauma centres, paediatric trauma centres and other medical rescue teams; (2) having cellular communication ensuring communication of the air medical rescue team with medical dispatchers, the voivodeship emergency medical coordinator and other emergency rescue teams and (3) having access to the Internet in places where air medical rescue teams are stationed.

The main contractor for tasks related to the implementation of sanitary transport in Poland is the Medical Air Rescue (MAR), in particular in the field of [53]:

- Emergency medical services (flights related to accidents and sudden illnesses and Providing assistance to the victims);
- Air sanitary transport performed outside the tasks of the State Emergency Medical Services system (transport of patients requiring medical care between medical entities);
- Medical air transport from abroad (e.g., transport of Polish citizens, victims of accidents or sudden illnesses, to Poland);
- Medical air transport outside the country.

The technical standard of sanitary transport is very similar in European countries. On the other hand, the organisational system is a derivative of national regulations regarding the functioning of healthcare, emergency medical services and national air transport. It also results from the degree of specialisation of hospitals and their resources, including unique diagnostic and life-saving equipment.

Hospital emergency departments are obliged, if necessary, to provide medical transport of the patient to the nearest medical entity providing healthcare services in the appropriate scope [54]. Air sanitary transport is performed between healthcare facilities in two modes [55]:

- Urgent (a rapid track procedure R)—a flight to help the patient in an urgent condition, requiring intensive supervision during the flight, when the delay may pose a threat to life or health;
- Scheduled (a slow track procedure S)—a planned air transport service for patients, performed by the medical air transport team in cases where the distance between the airports (registered in the “Aeronautical Information”) closest to the current and the target location of the patient exceed 250 km (according to the road maps) or in accordance with signed agreements.

According to official data of the Central Statistical Office (GUS) in Warsaw, in 2016, the total number of helicopter crews flights in Poland amounted to 8719 [56]. This number consisted of 7522 flights related to emergencies and illnesses and 1197 interhospital transports. The activity of medical air transport in Poland is increasing, which is confirmed by the latest data from 2020. The total number of flights in 2020 was 10,933, including 9636 flights related to accidents and emergencies and 1297 interhospital transports [57].

The location and operating conditions of helicopter bases are a particular determinant of the reliability of the medical air transport process. MAR has 21 permanent bases of the Helicopter Emergency Medical Service (HEMS) located throughout Poland, one seasonal base launched during the holidays and one transport team [53]. Duty in bases is carried out on in the 24 h system (four bases); from 7:00 to 19:00 (one base); from 7:00 to 20:00 (eight bases); and from 7:00, but not earlier than from sunrise, up to 45 min before sunset, but not later than until 20:00 (the remaining 9 bases, including the seasonal base, operate from 1 June to 5 September) [53].

Previous studies based on 911 hospital arrival intervals indicate that air medical transport is the fastest type of transport for patients for distances over 16 km, which contributes to its high reliability [58]. However, the total time it takes to transport patients by medical air transport compared to ground operations transport can minimally differ under optimal dispatch and transport conditions [59]. Medical air transport, depending on national legal regulations and legislation, faces various problems related to adaptation to specific patient needs. This applies recently to patients infected with the COVID-19 virus who are moved between hospitals [60]. In this case, reliability is critical to the operational continuity of the entire healthcare supply chain.

## 3. Material and Methods

In our research, we used the single case study method, which provides an in-depth analysis and a rich description [61]. The case study as an empirical research method can be exploratory, descriptive or explanatory and is used to study a contemporary phenomenon as it focuses on the dynamics of a case in the context of its real life [62]. A single case study allows for a deeper understanding of the explored topic, due to not only the possibility of a richer description of the phenomenon, but also the possibility of questioning the existing theoretical relations and exploring new ones as a consequence of conducting more detailed research [63]. Creswell et al. [64] emphasise that a case study is a qualitative approach in which the researcher examines one constrained system (case) or multiple constrained systems (cases) over a period of time, conducting detailed, in-depth data collection covering multiple sources of information (e.g., observations, interviews, audio-visual materials, documents and reports), and then prepares a report describing the case and its themes.

Our research was conducted at a highly specialised hospital with a hospital emergency department and a helipad for medical air ambulances. In the period of June–December 2021, we conducted in-depth expert interviews with people performing tasks in a group of 4 positions directly related to medical air transport. Partially structured interviews with specialists and entities were conducted, resulting in the identification of goals, tasks and conditions of healthcare supply chain management in the field of interhospital patient transfer using air ambulances. The research was carried out in 2 stages. In the first stage, the interviews featured questions divided into four groups:

1. Information on the expert.
2. Information on the participation of the expert in the studied process—his/her position and tasks performed.
3. Identification of problems and gaps by the expert in the course of previous practice.
4. Information on the system of relations between entities participating in the studied process.

The second round of interviews was carried out after the process map was made and included the following:

1. Questions regarding the correctness of the presented procedure according to the knowledge and practice of the expert and the analysis of the presented mapping.
2. Critical remarks on the solution presented.
3. Conclusions on the solution and the critical factors identified, as well as their verification.

This allowed for the implementation of the third stage consisting in the development of a supply chain map and analysis of the patient transfer process.

In the result of the logistics process map analysis, it was possible to identify reliability factors and develop solutions that could be used to ensure the reliability of the supply chain.

Among the people providing information were: the hospital manager, the manager of the department from which patients were transferred using MAR, the ward doctor who participated in the preparation of patients for transfer and the MAR doctor and MAR operations manager (Table 1). All these people have at least 5 years of work experience in the position and have repeatedly participated in procedures related to the use of MAR for medical purposes. The interviews were recorded and then transcribed. They were conducted before process mapping to obtain information about the patient transfer process and after mapping to verify it. In our research, we also used the active research method, because the research team included a member of the hospital management.

In total, as part of the interviews, we analysed 100 cases of interhospital air transport in the studied hospital. A comparative analysis of individual cases was carried out on the basis of orders for air transport. The disease entities for which patient transfers were carried out mainly concerned surgery and paediatric neurology, as well as adult neurological patients with brain haemorrhages and strokes. In this group, there were children with the following conditions: multiple-organ injuries, acute renal failure, head injuries and spinal injuries with neurological deficits. There were also cases of pregnancy pathologies (obstetric pathologies) threatening the life of the mother or child, as well as premature infants and babies born on schedule requiring transfer to a neonatal centre. The group of adult patients included cases of myocardial infarction qualifying the patient for immediate surgical intervention in a specialist hospital and scheduled cardiac transfers requiring surgical intervention in a specialist hospital. There were also cases of stroke and internal diseases requiring surgical intervention in a specialist hospital.

The second largest group comprised patients requiring a complex heart surgery or heart transplantation, as well as toxicology patients. Other cases of transfer included 2nd- and 3rd-degree burns exceeding 30% of the body surface area, including burns of the respiratory tract; severe organ failure; head injuries qualifying the patient for immediate surgical intervention in a specialist hospital; and acute diseases of the vascular system.

The smallest group included patients suffering from tropical and rare diseases.

Additional information was obtained from the analysis of hospital documents, MAR documents, regulations of the Ministry of Health, Quality Handbook of Best Practices, accreditation guidelines of the Government Quality Monitoring Centre and WHO recommendations.

The method used was process mapping, which requires a detailed analysis of the structure of the system, its goals (results), as well as the identification of entities and their responsibility for the processes and tasks carried out. Process mapping consists in building a model that shows the relationships between activities, people, data and objects involved in achieving a specific result and it is helpful in improving and redesigning business processes [65]. However, in relation to the healthcare supply chain, process mapping is used to a limited extent. Few previous studies relate to inventory management in the private healthcare sector [66], traceability of high-value products using RFID technology [67], network maturity [68]), drug distribution system [69], lean management practitioners and six sigma [66,69]).

According to Lambert and Cooper [70], the analysis of the supply chain management structure should take into account three closely related components: (1) the structure of the supply chain network, considered in the context of three elements, supply chain members, structural dimensions of the network and various types of process connections in the supply chain; (2) supply chain business processes—activities that give the customer a specific output value; and (3) supply chain management components, i.e., management variables that integrate and manage business processes throughout the supply chain. In this context, in the first stage, three main entities participating in the analysed logistics chain were identified:

- The hospital transferring the patient, ordering the patient’s transport, including the hospital head or authorised person, the hospital ward (doctors, the head of ward, nurses/paramedics), which orders transport of the patient, and the Hospital Emergency Department (HED) (medical and technical staff);
- The Medical Air Rescue, including a dispatcher and an air emergency team consisting of at least three people, including at least one professional pilot, a system doctor and a system paramedic or nurse;
- The hospital taking over the patient.

The analysed procedure was developed for the purposes of hospital accreditation carried out based on a set of accreditation standards approved by the Minister of Health, which is one of the important elements in the financing system. This procedure includes the following elements: legal basis, purpose, scope of responsibility, procedure (including patient qualifications and decisions, implementation rules—internal, safety rules and control activities) and appendices containing rules for ordering flights, transport order form and principles of radio communication.

## 4. Results

### 4.1. Case Description—Medical Air Transport Procedure

Our research was carried out in the HOSP1 voivodeship hospital located in a city with the population of nearly 80,000 people in the Lower Silesian Voivodeship in the period from June to December 2021. The founding body of the Hospital is the Local Government of the Lower Silesian Voivodeship. The main task of the Hospital is to provide inpatient and outpatient health services. The voivodeship hospital has 593 beds located in 18 wards and a dialysis unit (18 stations) and 22 specialist clinics.

In total, it employs about 190 doctors and 545 middle-level medical staff.

The basic areas of the hospital’s activity are inpatient services in the fields of hospital primary and specialist care; emergency assistance in the event of an accident, injury or sudden illness of patients not qualified for hospital treatment in urgent cases; therapeutic rehabilitation; and outpatient services in the fields of primary healthcare and specialist healthcare, laboratory and diagnostic activities and medical rehabilitation.

It is a typical multidisciplinary hospital, but due to typical economic constraints, it does not provide all (the full range of) medical services. This requires cooperation with specialist hospitals with appropriate, unique equipment and specialists, especially in rare specialties.

The hospital consists of three buildings with a multi-level structure connected with each other by internal and external passageways. It has three areas for parking lots and a system of internal roads throughout the area. The landing strip is located at a distance of several hundred metres from the main entrance. This makes it necessary to use sanitary transport, including an ambulance in the case of patient transfers. The location of the landing strip allows access not only to the main entrance, but also to each of the facilities constituting the hospital complex.

The hospital is partially computerised. The circulation of patient documents takes place generally inside the hospital with the use of an IT system of patient information, but the use of electronic signatures is excluded. Therefore, documents need to be printed—especially in the case of external transfers. This is why patients’ documents are transferred in paper form. However, archiving hospital documentation is already carried out in the IT system.

Inpatient round-the-clock hospital health services are provided in the fields of general surgery, vascular surgery, oncological surgery, orthopaedics and traumatology, paediatric surgery, paediatrics, obstetrics and gynaecology, neonatology, internal medicine, geriatrics, gastroenterology, cardiology, urology, nephrology, neurology, ophthalmology, otolaryngology, allergology, lung diseases, clinical oncology, emergency medicine, anaesthesiology and intensive care, neurosurgery, diabetology, medical rehabilitation and psychiatry.

Cases in which air medical transport is used in the studied hospital are all the instances of sudden deterioration of health or multiple-organ injuries that cannot be treated in a given hospital. In the HOSP1 hospital, they primarily concern children, because the hospital does not specialise in surgery, including trauma and paediatric neurology, hence, the need for transportation to the nearest trauma centre or hospital.

**Figure 1:**
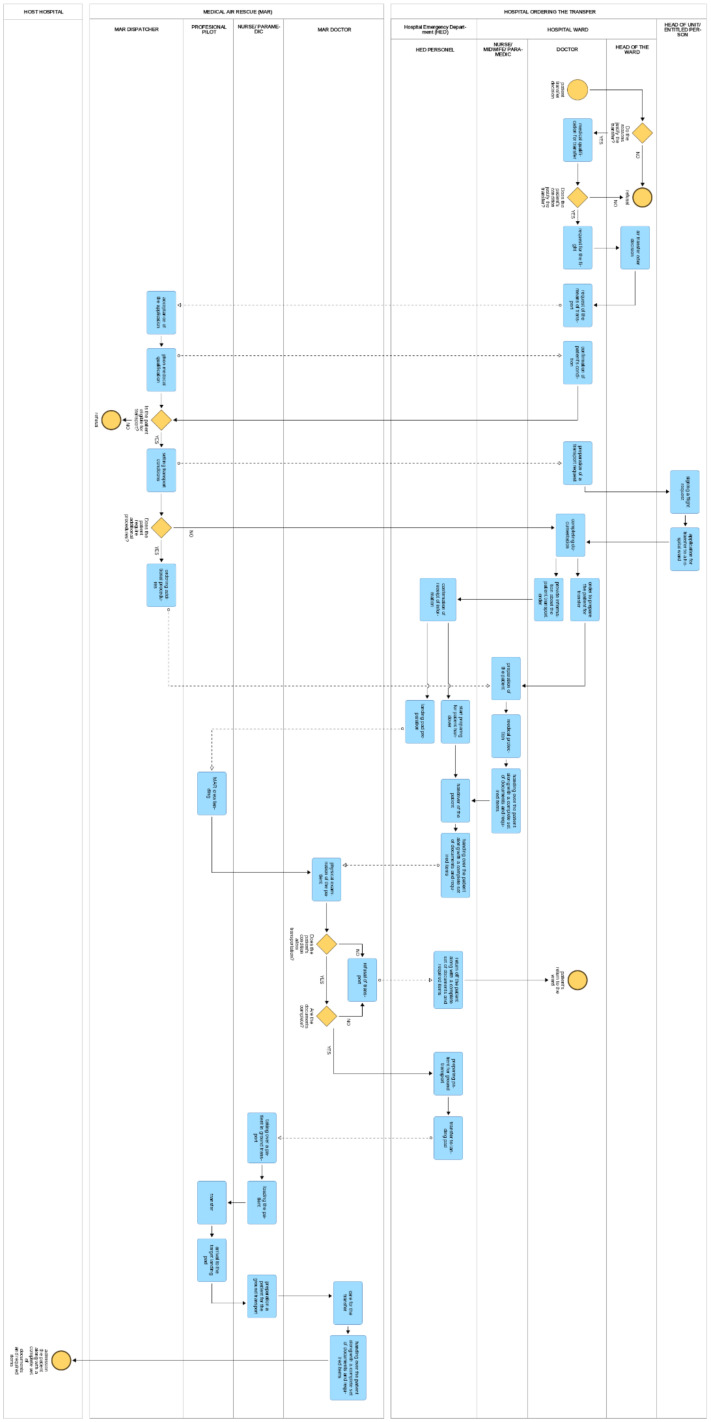
Procedure for the transfer of patients between hospitals in the case of qualifying the patient for transport by means of Medical Air Rescue.

Interhospital patient transfer is complex and involves several stages, including internal transfer between the hospital ward and the HED, transport of the patient from the HED to the helicopter, patient transit and the transfer to the target hospital. As part of these operations, a number of medical interventions (preparatory, stabilising and qualifying) are undertaken, which constitutes the premises for decisions on subsequent stages of the process. All operations must be adequately documented. The analysed patient transfer process was illustrated using the Adonis program (Figure 1). Combining semantic technologies with Management Information Systems based on Business Process Management Notation (BPMN) is useful in the public sector due to the large amount of information that can be shared [71]. The Adonis BPMN tool has previously been used in the healthcare sector to support discrete-event simulation [72].

The procedure was developed for the accreditation of hospitals, which is one of the important elements in the financing system. In accordance with the Act of 6 November 2008 on accreditation in healthcare, accreditation is aimed at confirming that the entity providing health services meets accreditation standards in the provision of health services and the operation of this entity. Accreditation is carried out by the Centre for Quality Monitoring in Healthcare in accordance with a set of accreditation standards approved by the Minister of Health [73].

Aspects related to patient movement are included in the section on the continuity of care (CC) and include the CC9 standard for the functioning of the patient transfer procedure [74]. This procedure consists of two specific procedures, CC9.1 “The hospital has procedures for transferring patients outside the hospital” and CC9.2 “The hospital has procedures for transferring patients between wards”, which should specify: (a) the method of transferring responsibility for the patient, as well as the type and scope of medical information provided during the transfer, and (b) the means of transport and the responsibility of persons providing care during the transfer of the patient [74]. This procedure includes the following elements: legal basis, purpose, scope of responsibility, course of action (including patient qualification and decisions, implementation rules—internal, safety rules, control activities) and appendices containing flight order rules, the MAR transport order form and the rules for conducting radio correspondence. Three main entities participate in the analysed logistics chain: the hospital transferring the patient, the Air Ambulance Service and the receiving hospital.

The effectiveness of interhospital air medical transport depends on the speed of implementation of transport orders by the operational centre of the Medical Air Rescue. Air medical transport is closely monitored in terms of the speed of patient collection and release to the receiving hospital. The shortest distance between hospitals was 110 km in a straight line for the 100 cases studied. The reliability of air medical transport depends on many independent factors, such as weather conditions, and factors related to operations management, such as fuel logistics. The COVID-19 pandemic has fundamentally changed the medical procedures associated with medical air transport. The changes concerned the need to equip helicopters with personal protective equipment to prevent the transmission of the SARS-CoV-2 virus in the case of COVID-19 patients. The disinfection of helicopters after the completion of patient transport between hospitals also became an issue. This is a considerable problem, as helicopter cleaning procedures take from 2 to 3 h.

COVID-19 has made it necessary to add additional tests related to determining the patient’s condition, but testing positive for COVID-19 does not exclude the patient from the transfer. It does, however, cause the necessity to provide additional protection for the staff, flight service and their equipment. This reduces the availability of transfer and increases the number of activities to be performed by process participants to include further actions aimed at protecting participants and the patient. They concern the isolation and limitation of the spread of the virus but do not affect the shape of the model. MAR transports patients in different epidemic states with different disease entities, including carriers of various infectious, tropical diseases and rare diseases. For such situations, MAR and other medical entities have appropriate internal procedures in place regardless of the current epidemic situation.

### 4.2. Identification of Key Factors

The analysis of the healthcare supply chain in the HOSP1 hospital covering patient flow between hospitals by means of the air medical transport led to the identification of thirteen factors that affect reliability. These factors were indicated by all participants of expert interviews. These factors are environmental (geographical, climatic), systemic (legal), technical and technological, organisational (communication systems, human resources, documentation), concerning the patient’s condition and temporal. In particular, the following factors are relevant:

1. Legal—including regulations at the system level (laws, regulations, standards);
2. Administrative—at the sectoral/specialist and internal level—of hospitals;
3. Geographical and climatic (weather, terrain, elements of the area: lakes, rocks, trees, marshes);
4. Technical—flight range; distance; conditions of airport infrastructure, e.g., traction networks; structures hindering landing (e.g., silos or windmills);
5. Infrastructural—dedicated to patients’ air transport (landing pads, runways, landing pad lighting, technical equipment of the landing pad—direction indicators to fly in difficult conditions such as fog, wind protections), the number and quality of bases, the number and availability of service and repair systems;
6. Means of transport—quantity, quality, equipment, availability, rate of wear and tear;
7. Staff—the availability and qualifications of staff (implementing activities in the MAR and organisational units of the hospital), health and psychophysical conditions of staff (admission to flying);
8. Patient’s condition entitling to and allowing transport (specific medical, administrative and organisational indications)—patients expected to not survive the transport with a sudden cardiac arrest, patients in the second phase of childbirth and patients who may pose a threat to the mission’s safety are not eligible for air transport [75];
9. Documentation—completeness (at the stage of requesting, medical qualification, patient’s medical documents, referral to the hospital), confirmation of effectiveness (ensuring the continuity of care and appropriateness of procedures followed), technical standard of documentation, applied IT and information systems, interoperability—system compatibility;
10. Being equipped with medications and medical supplies necessary to ensure patient safety, continuity of treatment and saving lives (the standard of equipping MAR with medications may be different from the standard applicable to hospital or rescue units);
11. Communication—communication speed determined by the technical condition of equipment, device compatibility, standardisation of messages, a unified language code and specialised terminology; prioritisation of requests (taking into account the urgency of the situation, the conditions of the transfer), complexity of the communication system (a multitude of entities and arrangements at the internal organisational level—hospital and inter-organisational (between hospitals, between the hospital and the MAR base including the dispatcher, between the hospital/HED and the MAR crew)), individualisation of relations between system participants;
12. Human factor—personal predisposition, psychophysical condition, ability and willingness to make decisions under pressure (threat), reaction to stress, reaction speed and involvement of staff accompanying the patient at all stages of the process;
13. Time—related to the needs of the patient (medical aspects, patient’s condition and development of the disease), referring to the organisation of flow: time necessary to prepare the patient, time of flight, time related to readiness of service (of all groups of entities, including the receiving hospital), operating time and the time to reach the target parameters.

An important logistic factor determining the duration of the process is achieving the appropriate take-off readiness. According to Polish standards, the parameters of the time of arrival to the place of an air ambulance team depend on the radius of operation, and therefore [76]:

- The readiness to take off is 3 min during the day and 15 min at night—when the radius of operation is less than or equal to 60 km,
- The readiness to take off is 6 min during the day and 30 min at night—when the radius of operation is greater than 60 km and less than or equal to 130 km,
- The readiness to take off is 15 min during the day and 30 min at night—when the radius of operation is greater than 130 km.

According to the Medical Air Rescue, the medical air transport team in the rapid track procedure is ready to take off in 60 min, while in the case of an emergency Medical Air Rescue team, the readiness depends on the procedure and time of day and is accordingly as follows: in a rapid track procedure, during the day—15 min and at night—15 min, and in a slow track procedure during the day—15 min and at night—30 min (with an agreement from the Ward Director) [55].

## 5. Discussion

The indicated factors resulting from the conducted analyses constitute the main group of determinants of the reliability of the medical supply chain transport in the healthcare system. Existing regulations form the general framework for implementing the patient air transport process. In the context of dynamic changes in the environment and the functioning of the healthcare supply chain healthcare, it is necessary to take actions aimed at improving these solutions.

The analysis of the presented process leads to the conclusion that in order to ensure reliability, it is necessary to manage the process in an integrated manner at the system, inter-organisational and intra-organisational levels. Patient air transport is one of the key challenges in providing adequate medical care to patients, maintaining its continuity and the availability of highly specialised medical procedures. Therefore, this process requires proper planning of procedures, standards and rules of conduct, organising human resources, equipment and infrastructure, coordinating the processes of patient interhospital transfer (decision-making, inter-organisational cooperation) and controlling the effectiveness of transfers.

Shaping the right relationships with patients, taking into account their individual needs and conditions, plays a particularly significant role in the supply chain. They are the result of, among other things, the relationships created by all system participants.

At the system level, regulations determining quality, staff (qualifications and competences) and infrastructure (technical) requirements as well as appropriate financing mechanisms are of key importance. At the inter-organisational level, collaboration is vital, taking into account communication strategies, relationships and processes, in particular those that focus on care coordination [77].

There is a need for a comprehensive approach to care based on the concept of transitional care, covering a broad set of activities to ensure coordination and continuity of healthcare when patients are transferred between different locations or different levels of care at the same location, including logistical arrangements, patient and family education and coordination among the healthcare professionals involved in patient flow [78].

To ensure efficient, successful patient transfer and immediate assumption of responsibility for patient care, timely, accurate and sufficient communication of clinical information among service providers is crucial [79].

At the organisational level, there are a number of relationships between healthcare professionals and patients (including those of highly individualised nature) that may be direct sources of risk of ineffective care or a threat to patient safety. In general, in the interhospital transport of patients, the range of risk factors of adverse events is wide and may include (a) technical factors related to the equipment and its use, (b) human factors related to the transport team (including lack of training or lack of supervision), (c) collective factors related to the organisation of transport, including insufficient communication and coordination between referring and receiving teams, and (d) patient-related factors (including clinical instability) [16].

The created procedure is based on generally applicable Polish laws, modelled on European regulations. To create it, we used the triangulation of methods. The model presented in the single case study presents a universal subject structure (hospital + Hospital Emergency Department (HED) + MAR + receiving hospital). This proves that our procedure can be used in other hospitals not only in Poland. However, depending on the technical potential of the hospital, we may observe slight modifications to the procedure.

The reliability of the supply chain, including interhospital patient flow implemented by the air medical transport system, should be analysed in terms of three basic criteria: effectiveness, timeliness and patient focus (health status). In this context, reliability is an unimpeded timely transfer of the patient, carried out without deterioration of the patient’s condition and confirmed by appropriate and complete documentation. The specificity of the health services sector means that reliability is a category strictly associated with the focus on the patient’s needs (patient-centredness) and it requires an integrated action (at the system, inter-organisational and patient—professional levels) focused on maintaining the continuity of care.

Improving the logistics process requires a clear definition of the roles and responsibilities of all entities involved in the healthcare supply chain, standardisation of processes related to consultation (patient qualification), medical documentation, test results, information flow and communication aspects. For this purpose, there is a need for electronic medical records while maintaining interoperability between organisations.

## 6. Conclusions

Our research has proven that process mapping in the healthcare supply chain allows us to move from the legislative (directive) level to the operational and tactical (executive) level. This is especially important for understanding the specifics of the operation of the supply chain in which the links are independent or remain in inaccurate relationships that hinder the coordination of flows. The presented research results allow us to understand the course of the process and the role of individual entities participating in it. A critical element for the whole process is the patient’s state of health from the moment they are qualified for the procedure. Given that the transfer concerns sick people, their state of health may deteriorate so much that it will not be possible to carry out the transport. In a situation of deterioration of the patient’s health, even the doctor of the MAR crew may refuse to take the sick person for whom the helicopter arrived. This partially stochastic nature of the healthcare supply chain means that reliability plays a key role in ensuring patient safety. This safety is covered by MAR procedures and regulations. Our research points to two main types of factors influencing the reliability of the supply chain in the case of interhospital medical air transport. The first concerns the MAR itself, which has limited financial resources to meet all the needs identified by hospitals. Limited financial possibilities force a very thorough analysis of each case, and if there are transfer options other than air medical transport, the type of patient transfer between hospitals is changed. The implementation of planned tasks, i.e., the transfer of patients by air transport, encounters bottlenecks due to unplanned urgent MAR transports. This is a resource constraint for medical air transport between hospitals. The second type of factors is related to the dynamics of changes in weather conditions and the time of day. This limits the availability of planned patient transfers.

This article may, in particular, contribute to the analysis of the decision-making process within the patient transport system involving medical air ambulance services. Our results highlight the importance of reliability, which is a pillar of air transport processes in patient transfer and is critical to the effectiveness of patient treatment. The interdisciplinary approach contributes to the development of public health and interhospital logistics in the operational dimension, and it contributes to the development of research in the literature on healthcare logistics. Examination of patient transport from the perspective of the supply chain concept allows for a multi-faceted analysis and highlights the multitude of challenges resulting from the complexity and difficulty of solving logistics problems. In this work, a case study was conducted using real data. The particularly risky process of patient flow with the participation of the air medical team was empirically investigated, which led to the identification of the key reliability factors of this process. Our research allowed the hospital to understand the complexity of the factors affecting the reliability of the healthcare supply chain and to improve some decision-making processes. This was made possible by the use of triangulation of research methods, as a result of which the processes shaping the reliability of the supply chain in the hospital were fully mapped.

The proposed process mapping method is an analytical tool that can be used to implement decision-making processes at the operational level in individual hospitals. It is an effective instrument that allows for the control and improvement of planned and implemented quality procedures in hospitals. The changes proposed in the analysed logistics chain can contribute to ensuring the reliability of the entire healthcare supply chain at the organisational and inter-organisational levels.

The authors recognise the potential limitations of the designed research procedure, in particular regarding the selection of the research subject, the scope of the analysis and the failure to identify all the factors determining the reliability of patient air transport processes. The method of using a single case study used has allowed to develop a procedure for interhospital transport by air medical transport in the selected hospital. The applicability of the selected research method (case study) is limited by the situational context related to the specificity of the functioning of national healthcare systems and transport institutions defining the framework for patient transport processes. Our observations have limitations due to the fact that they were formulated on the basis of research carried out in one public regional hospital. However, the identified factors affecting the reliability of HSC and the procedure for transporting patients between hospitals can serve as a reference for other hospitals, especially in the context of understanding the complexity of the factors influencing the management of operations in hospitals. However, in order to formulate recommendations for the reliability of processes carried out at the inter-organisational, systemically regulated level, it is necessary to expand the scope of research facilities (hospitals, medical air transport units).

In the future, our research can be extended to include issues related to communication processes characterised by a high level of complexity due to the multiplicity of participants in interhospital transfers. A diverse range of information needs in healthcare supply chain management creates the necessity for coordination of information flows. In this context, it is appropriate to focus further research on coordination activities in support of decision-making at the operational level.

## Figures and Tables

**Table 1:** Characteristics of the interviewed experts.

Interviewer	Responsibilities with Regard to the Air Transport of Patients (Medical Specialisation)	Number of Meetings and Total Duration of Interviews
The hospital manager	Verification and approval of the order	(2) Total time—1:15
The manager of the department from which patients were transferred using MAR	Verification and approval of the order, transfer of the decision to the MAR	(4) Total time—3:10
The ward doctor who participated in the preparation of patients for transfer	Qualifying the patient for the procedure, ordering the preparation of patient documents, drawing up a transport order	(10) Total time—3:25
The MAR doctor	Verification of the patient’s condition and the final decision on the order implementation	(2) Total time—1:10
MAR operations centre manager	Acceptance of the order, qualification and decision on implementation	(2) Total time—1:20

## Data Availability

Not applicable.
